# Organic Anion Transporter 5 Renal Expression and Urinary Excretion in Rats with Vascular Calcification

**DOI:** 10.1155/2013/283429

**Published:** 2013-10-02

**Authors:** María Herminia Hazelhoff, Romina Paula Bulacio, Adriana Mónica Torres

**Affiliations:** Area Farmacología, Facultad de Ciencias Bioquímicas y Farmacéuticas, Universidad Nacional de Rosario, CONICET, Suipacha 531, 2000 Rosario, Argentina

## Abstract

It has been described renal damage in rats with vascular calcification. The organic anion transporter 5 (Oat5) is only expressed in kidney, and its urinary excretion was proposed as potential early biomarker of renal injury. The aim of this study was to evaluate the Oat5 renal expression and its urinary excretion in an experimental model of vascular calcification in comparison with traditional markers of renal injury. Vascular calcification was obtained by the administration of an overdose of vitamin D_3_ (300,000 IU/kg, b.w., i.m.) to male Wistar rats. Oat5 urinary abundance was evaluated by Western blotting. Traditional markers of renal injury, such as creatinine and urea plasma levels, urinary protein levels, and urinary alkaline phosphatase (AP) activity, were determined using commercial kits. Histology was assessed by hematoxylin/eosin staining. Oat5 renal expression was evaluated by Western blotting and by immunohistochemistry. An increased expression of Oat5 in renal homogenates, in apical membranes, and in its urinary excretion was observed in rats with vascular calcification. The traditional parameters used to evaluate renal function were not modified, with the exception of histology. It is possible to postulate the urinary excretion of Oat5 as a potential noninvasive biomarker of renal injury associated with vascular calcification.

## 1. Introduction

Calcium overload in vascular smooth muscle is an active process which not only progresses with aging but also is associated with various age-related vascular diseases such as atherosclerosis, hypertension, and cardiac valve disease [1–3]. An experimental model that produces an increase in arterial wall calcium content is achieved by the administration of an overdose of vitamin D_3_ [4–8]. In this model, calcium overload is observed in the arterial wall, in the heart, and in the kidneys leading to the impairment of their function [2]. Morphological and functional alterations were described in the kidney [5–8]. 

Organic anion transporters (Oats in animals/OATs in humans, subfamily of Slc22 drug transporters) mediate transport of endogenous and exogenous organic anions (OA) and play a major role in the uptake and/or secretion of OA in cells of various mammalian organs, mainly in the liver and in the kidneys. The organic anion transporter type 5 (Oat5, Slc22a19) from rats and mice has been cloned and characterized as an organic anion/dicarboxylate antiporter [9–12]. This protein is exclusively expressed in the kidneys and is located at the brush border membrane of the proximal tubule straight segment S3, in the outer stripe of the outer medulla and in the juxtamedullary cortex [10, 12]. It transports dehydroepiandrosterone sulfate, estrone-3-sulfate, and ochratoxin A [9–12]. Rat Oat5 interacts with *α*-ketoglutarate, with succinate, and with chemically heterogeneous anionic compounds, such as diuretics, nonsteroidal anti-inflammatory drugs, *β*-lactam antibiotics, and bromosulfophthalein [10].

Our group was pioneer in detecting Oat5 in urine and proposing its urinary excretion as an early indicator of renal injury [13–15]. We have recently described a dramatic urinary increase of Oat5 in the presence of bilateral mild subclinical ischemia and bilateral ischemia with acute kidney injury suggesting that Oat5 might be a potential early noninvasive marker of this pathology [13]. Moreover, the urinary excretion of Oat5 has also been suggested as an early indicator of nephrotoxicity induced by mercury and by cisplatin [14, 15].

The aim of this study was to evaluate Oat5 renal expression and its urinary excretion in renal damage associated with vascular calcification in comparison with traditional markers of renal injury, such as histology, creatinine and urea plasma levels, urinary protein levels, and urinary alkaline phosphatase (AP) activity. 

## 2. Materials and Methods

### 2.1. Experimental Animals

Adult male Wistar rats (310–360 g; aged 110–130 days) were used. Animals had free access to a standard laboratory chow and tap water and were housed in a constant temperature and humidity environment with regular light cycles (12 h) during the experiment. All experiments were conducted according to NIH Guide for the Care and Use of Laboratory Animals. For surgical procedures rats were anesthetized with sodium thiopental (70 mg/kg, b.w., i.p.).

**Figure 1:**
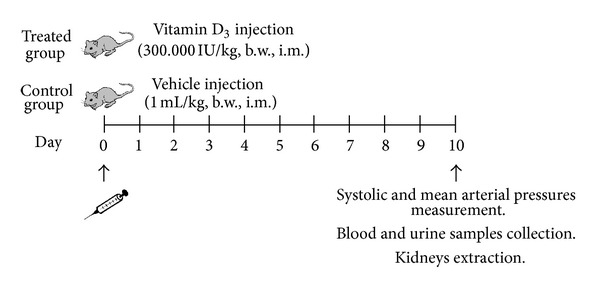
Diagram of study design. Animals were randomly divided into two groups: control and treated rats. Treated rats were injected with a single overdose of vitamin D_3_ (300,000 IU/kg, b.w., i.m.) 10 days before the experiments in order to achieve an experimental model of vascular calcification as previously described [4–7]. Vitamin D_3_ solutions were prepared in corn oil. Control animals received an identical volume of corn oil by i.m. injection (1 mL/kg).

Animals were randomly divided into two groups: control and treated rats. Treated rats were injected with a single overdose of vitamin D_3_ (300,000 IU/kg, b.w., i.m.) 10 days before the experiments in order to achieve an experimental model of vascular calcification as previously described [2, 4–8]. Vitamin D_3_ solutions were prepared in corn oil. Control animals received an identical volume of corn oil by i.m. injection (1 mL/kg). The diagram of study design is shown in Figure 1.

Two different sets of experimental animals were employed.

- One set of rats was used for histopathological and immunohistochemical studies in kidneys and histochemical calcium assay in abdominal aorta (*n* = 4 for each experimental group).
- Another set of animals was used for measurement of arterial pressures. Then, the rats were anesthetized as previously described, blood was collected from the heart in heparinized syringe, and urine was collected from the urinary bladder with a syringe immediately after opening the abdominal cavity. Urine samples were obtained for biochemical assays and for detection of Oat5 by Western blotting, and blood samples were used for biochemical parameters assays (*n* = 4 for each experimental group). Finally, kidneys were removed for preparation of homogenates and apical membranes for Western blotting studies, and abdominal aorta was extracted for analyzing tissue calcium levels (*n* = 4 for each experimental group).

### 2.2. Measurement of Arterial Pressure

Systolic and mean arterial pressure were measured in both control (*n* = 4) and treated rats (*n* = 4) with a Harvard indirect rat tail blood pressure monitor (Harvard Apparatus, Millis, MA, USA) connected to a Harvard student oscillograph, as previously described [5–8]. 

### 2.3. Vascular Calcium Analysis

Calcium levels in abdominal aorta were evaluated by a spectrophotometric technique and by histochemistry employing von Kossa staining. For spectrophotometric studies abdominal aorta was removed, weighed, and then heated to constant dry weight for 48 h at 120°C. Dry samples were dissolved in nitric acid (14 mol/L) and left for 72 h at room temperature. Samples were then centrifuged (2000 ×g, 10 min, and at room temperature). Strontium nitrate was added and calcium was measured by atomic absorption spectrophotometry, as described by Quaglia et al. [5–7]. For calcification studies using von Kossa method, abdominal aorta was dissected and rinsed in saline solution and immersed in periodate-lysine-paraformaldehyde solution (0.01 M NaIO_4_, 0.075 M lysine, and 0.0375 M phosphate buffer, with 2% paraformaldehyde, pH: 6.20), at 4°C overnight. Then, the tissue was embedded in paraffin. Paraffin sections were cut. After deparaffining, the sections were hydrated and exposed to 5% silver nitrate under ultraviolet light for 60 min. After washing in 5% sodium hyposulfite for 3 min, the sections were counterstained with 0.01% nuclear fast red for 5 min and examined under a light microscope similar to that previously described by Han et al. [16].

### 2.4. Biochemical Determinations

Urine samples (centrifuged at 1000 ×g for 10 min to remove cell debris) were used for analyses of AP activity, proteins concentration, and creatinine concentration. Blood was extracted by cardiac puncture, and blood plasma was separated by centrifugation (1000 ×g for 10 min). These samples were used to assay calcium levels, urea concentration, and creatinine concentration. 

All parameters were determined spectrophotometrically using commercial reagent kits (Wiener Laboratory, Rosario, Argentina).

### 2.5. Histopathological Studies

Histopathology of kidneys was performed after fixing them in 10% neutral buffered formaldehyde solution for 4 h and embedding them in paraffin. Then 4 *μ*m thick sections were processed for routine staining with hematoxylin and eosin.

### 2.6. Immunohistochemistry

The immunohistochemistry technique was performed as previously described [13–15]. Kidneys from both experimental groups of rats were briefly perfused with saline, followed by perfusion with periodate-lysine-paraformaldehyde solution (0.01 M NaIO_4_, 0.075 M lysine, and 0.0375 M phosphate buffer, with 2% paraformaldehyde, pH 6.20), through a cannula inserted in the abdominal aorta. Kidney slices were immersed in periodate-lysine-paraformaldehyde solution at 4°C overnight. The tissue was embedded in paraffin. Paraffin sections were cut. After deparaffining, the sections were incubated with 3% H_2_O_2_ for 15 min (to eliminate endogenous peroxidase activity) and then with blocking serum for 30 min. These sections were then incubated with noncommercial polyclonal antibody against Oat5 (diluted 1 : 100) overnight at 4°C. The specificity of Oat5 antibody has been described elsewhere [10]. The sections were rinsed with PBST. Then, the sections were incubated with horseradish peroxidase- (HRP-) conjugated secondary antibody against rabbit immunoglobulin for 1 h. In order to detect HRP labeling a peroxidase substrate solution with diaminobenzidine (0.05% diaminobenzidine in PBST (80 mM Na_2_HPO_4_, 20 mM NaH_2_PO_4_, 100 mM NaCl, and 0.1% Tween 20, pH 7.50) with 0.05% H_2_O_2_) was used. The sections were counterstained with hematoxylin before being examined under a light microscope. 

Controls using preimmune serum and antiserum absorbed with excess synthetic peptide or omission of primary or secondary antibody revealed no labeling.

### 2.7. Preparation of Apical Membranes from Kidney

Apical membranes were isolated from kidneys by Mg/EGTA precipitation as previously described [13–15]. The kidneys were removed, minced, and homogenized in 30 g/100 mL of ice-cold 50 mM mannitol, 2 mM Tris HCl buffer (pH 7.10), 5 mM EGTA, and 1 mM PMSF for 5 min at top speed in a* Glas-Col *homogenizer. From this preparation we obtained total homogenates, and aliquots were taken and stored at −80°C until use.

Then, MgCl_2_ was added to the remaining homogenate to a final concentration of 12 mM, and the mixture was stirred in an ice bath for 15 min. The homogenate was then centrifuged (3000 ×g, 15 min, and 4°C). The supernatant was carefully decanted and centrifuged again at 28,000 ×g for 40 min at 4°C. The pelleted material representing brush border membranes was resuspended in 50 mM mannitol, 10 mM HEPES-Tris (pH 7.50), and 1 mM PMSF and centrifuged for 15 min at 800 ×g at 4°C. The supernatant was finally centrifuged for 45 min at 28,000 ×g. The brush border membranes pellets thus obtained were resuspended in 50 mM mannitol, 10 mM HEPES-Tris buffer (pH 7.40), and 1 mM PMSF. Aliquots of the membranes were stored immediately at −80°C. Each preparation represented renal tissues from four animals. The enrichment and purity of these membranes were comparable to those previously reported [13–15]. Protein quantification of samples was performed using the method of Sedmak and Grossberg [17]. 

### 2.8. Electrophoresis and Immunoblotting

Homogenates (35 *μ*g of protein), apical membranes (25 *μ*g of protein), and urine (10 *μ*L) samples were boiled for 3 min in the presence of 1% 2-mercaptoethanol/2% SDS. Samples were applied to an 8.5% polyacrylamide gel, separated by SDS-PAGE, and electroblotted to nitrocellulose membranes. To verify equal protein loading and transfer between lanes, Ponceau Red was used as previously described [14, 15, 18]. The nitrocellulose membranes were incubated with 5% nonfat dry milk in PBST for 2 h. After being rinsed with PBST, the membranes were incubated overnight at 4°C with a rabbit polyclonal antibody against rat Oat5 (at a dilution of 1 : 800). The specificity of Oat5 antibody has been described elsewhere [10]. The membranes were incubated for 1 h with a peroxidase-coupled goat anti-rabbit IgG (Bio-Rad; Hercules, CA) after further washing with PBST. Blots were processed for detection using commercial kit (Pierce ECL Western Blotting Substrate; Thermo Scientific, Rockford, IL, USA). A densitometric quantification of the Western blotting signal intensity of membranes was performed. For densitometry of immunoblots, samples from treated rat kidneys were run on each gel with corresponding control kidneys. The abundance of Oat5 in the samples from treated animals was calculated as percentage of the mean value of control rats for that gel.

### 2.9. Materials

Chemicals were purchased from Sigma (St. Louis, MO) and were of pure analytical grade. The polyclonal antibody against Oat5 employed in both immunohistochemistry and immunoblotting technique was courteously given by Professor H. Endou and Professor N. Anzai (Department of Pharmacology and Toxicology, Kyorin University School of Medicine, Tokyo, Japan).

### 2.10. Statistical Analysis

The statistical analysis was performed using the unpaired Student *t*-test. When variances were not homogeneous, Welch's correction was employed. *P* < 0.05 was considered statistically significant. Data are expressed as means ± standard error (SEM). For these analyses, GraphPad software was used. 

## 3. Results

As it is shown in Table 1, systolic arterial pressure (SAP) and mean arterial pressure (MAP) were increased in rats treated with vitamin D_3_. In contrast, body weight, kidney weight, and its ratio were not modified with the treatment. Plasma calcium, urea, and creatinine levels did not change in treated rats when compared with control ones. Total calcium levels (*μ*mol/g dry weight) were significantly increased in aorta from treated animals. 

**Figure 2:**
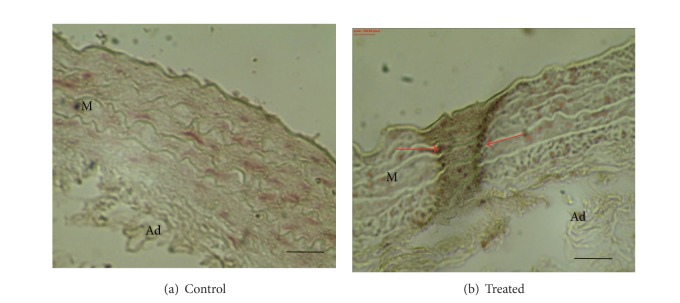
Determination of calcium load in abdominal aorta from control (a) and treated (b) rats. These pictures are representatives of samples obtained from four animals from each experimental group. Sections were stained according to the von Kossa method. von Kossa staining demonstrated the absence of calcification in control blood vessel whereas treated ones showed calcification. Calcified nodules are marked with arrows. M indicates media; Ad indicates adventitia. Bars: 40 *μ*m.

Areas of medial calcification were detected in von Kossa-stained sections as deposits of brown-black material in aortas from rats with vascular calcification (Figure 2).

**Figure 3:**
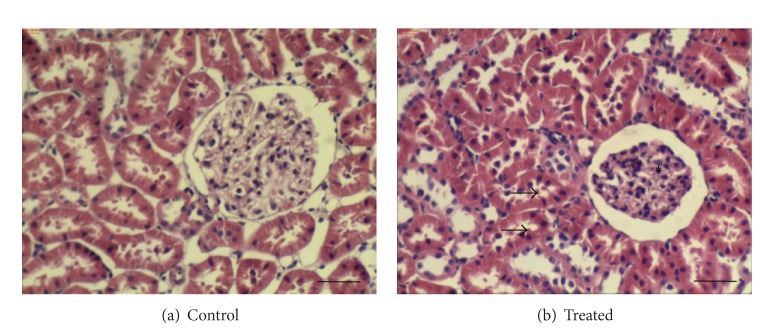
Optical microscopy photos of kidney histology from control and treated (vitamin D_3_ 300,000 IU/kg, b.w., i.m.) animals. These pictures are representatives of samples obtained from four animals from each experimental group. Renal histological studies revealed tubular alteration with vacuoles in the cytoplasm (arrows), glomeruli of reduced size (asterisks) in kidneys from rats with vascular calcium overload. Bars: 40 *μ*m.

Histopathological studies assessed by hematoxylin/eosin staining revealed tubular alterations with vacuoles in the cytoplasm, glomeruli of reduced size, and no morphological changes in the interstitium of kidneys from rats with vascular calcium overload (Figure 3). These modifications are consistent with those previously described for this experimental model [4, 7, 8].

**Figure 4:**
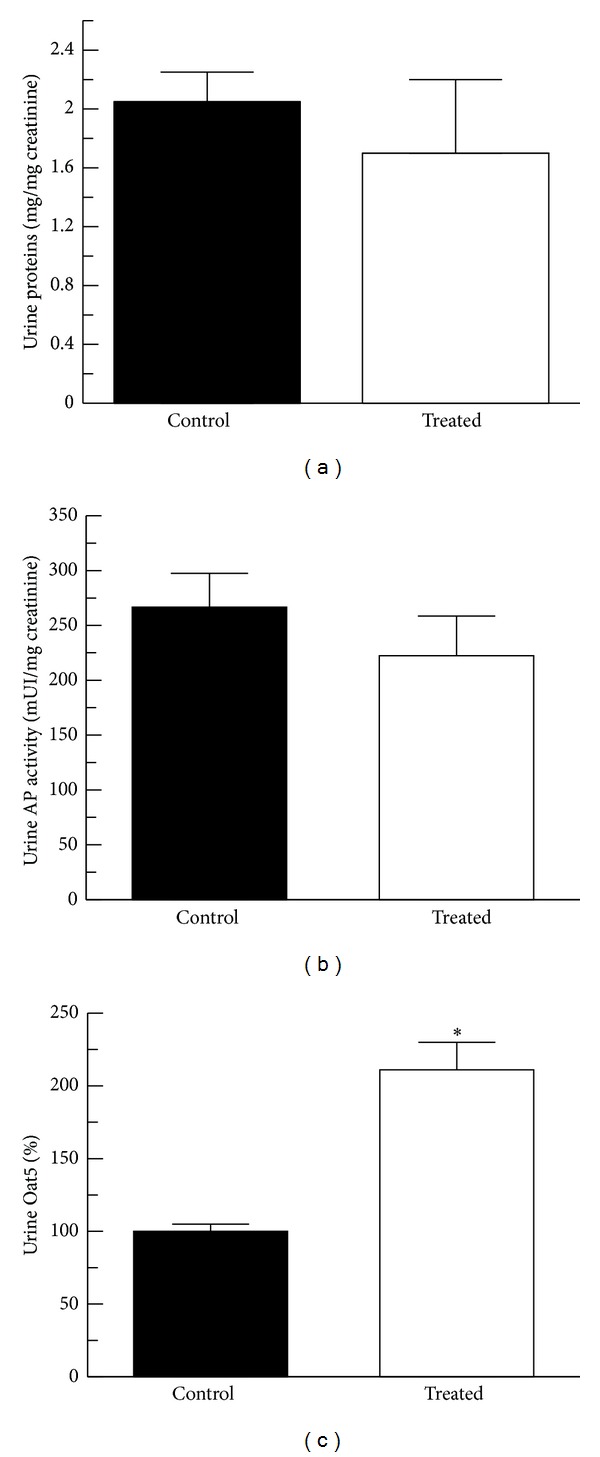
Total protein levels (a), alkaline phosphatase (AP) activity (b), and Oat5 abundance (c) in urine from control (*n* = 4) and treated (vitamin D_3_ 300,000 IU/kg, b.w., i.m., *n* = 4) animals. Total urine protein levels and AP urinary activity were determined using commercial kits and related to urinary creatinine concentrations to correct them for variations in urine production. Oat5 abundance was determined by Western blotting using a noncommercial antibody as described in Section 2. Densitometric quantification of Oat5 immunoblotting from urine is expressed as arbitrary units also related to urinary creatinine concentrations, and the mean of the control levels was set as 100%. Results are expressed as mean values ± SEM. *P < 0.05.

Other traditional parameters of renal function as total proteins and AP activity in urine were evaluated. Total proteins in urine related to urinary creatinine levels were not modified with the vitamin D_3_ treatment as shown in Figure 4(a). AP activity in urine (also normalized to urinary creatinine concentration) was not changed in the treated group compared with the control group (Figure 4(b)).

The concentration of proteins and AP activity in urine were related to urinary concentration of creatinine in order to correct variations in urine production as previously described for total proteins, transporters, and enzymes [13–15, 19–23]. Normal physiological variations in urinary water excretion can dilute or concentrate such proteins, transporters, and enzymes; therefore individual measurements of these parameters are insufficient. Creatinine is excreted in urine at relatively constant rates; hence it can be used to normalize urinary excretion of a particular parameter. Oat5 abundance in urine was also related to urinary creatinine concentration as described above.  Figure 4(c) shows that Oat5 abundance in urine samples was significantly higher in rats that received vitamin D_3_. 

**Figure 5:**
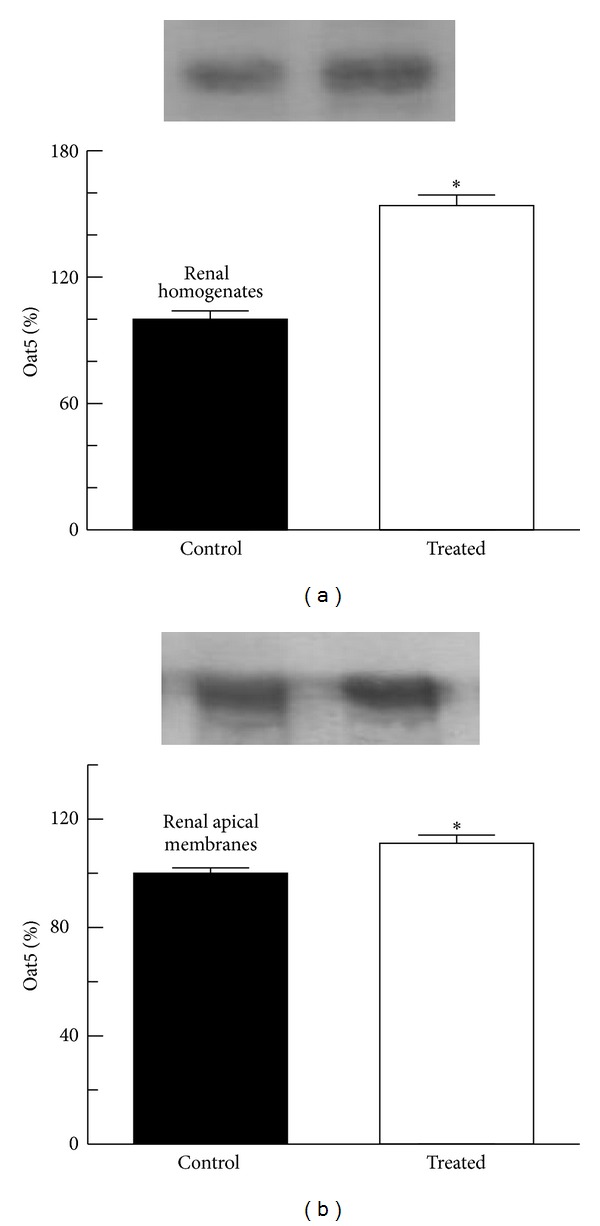
Oat5 abundance in renal homogenates (a) and apical membranes (b) from kidneys of control and treated (vitamin D_3_ 300,000 IU/kg, b.w., i.m.) rats. Proteins were separated by SDS-PAGE and blotted onto nitrocellulose membranes. Oat5 was identified using a noncommercial antibody as described in Section 2. The mean of the control levels was set as 100%. Each column represents the mean ± SEM from experiments carried out in four animals for each experimental group. *P < 0.05.

As shown in Figure 5, there was a significant increase in Oat5 abundance in homogenates and in apical membranes from vitamin D_3_ treated rats compared to those from control animals.

**Figure 6:**
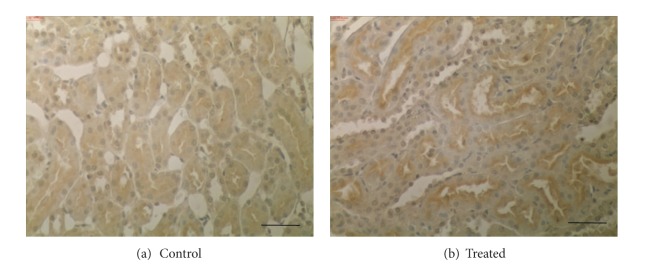
Immunohistochemistry for Oat5 in kidneys from control and treated (vitamin D_3_ 300,000 IU/kg, b.w., i.m.) animals. Serial sections from each rat kidney were stained using a noncommercial anti-Oat5 antibody. In kidneys from treated group a marked increase in the Oat5 staining was observed. These figures are representatives of typical samples from four rats for each experimental group. Bars: 40 *μ*m.

Immunohistochemistry studies revealed Oat5 labeling associated with the apical membrane domain in proximal tubule cells as previously described [10, 13–15]. In vitamin D_3_ treated animals a strong increase in Oat5 labeling was observed (Figure 6). These results corroborated the data obtained by Western blotting.

## 4. Discussion

Even if, in the last years, several breakthroughs in the knowledge of renal pathologies have been made, these have not been successful regarding the decrease of high rates of mortality associated with them. The lack of safe and reliable markers to detect diseases on their onset delays the start of a treatment [24, 25].

A biomarker can be defined as any plausible parameter being objectively evaluated in a patient as an indicator of normal biological, pathologic, or therapeutic response processes. A biological marker should have the following ideal characteristics: easily measurable on an accessible fluid, stability in the said fluid, detectable on early stages before the traditional kidney injury markers that are modified, being a specific injury marker of certain area of the kidney, and having the value to predict its evolution [26, 27].

Low molecular weight proteins, enzymes, urinary antigens, and transporters, which have been detected in urine in early stages of kidney damage, have been used as biomarkers of renal disease [28–30]. Regarding transporters, we can mention that aquaporin 2 has been detected in rat and human urine and it has been used as a marker for different pathologies [31, 32] and that isoform 3 of the Na^+^-H^+^ exchanger NHE3 has been proposed as acute renal failure marker [33].

Organic anion transporter type 5 (Oat5/OAT5) has been studied in rat, mouse, and human organs, but the available data indicate that the rodent and human genes are not orthologs and code for different proteins. Oat5 was first isolated from a mouse cDNA library in 2004 [9]. Subsequently, Oat5 was cloned from a rat kidney cDNA library [10]. Human OAT5 was cloned in 2001 [34]. However, no functional data has yet been obtained for the hOAT5 clone, and it is now believed that it is not the human orthologue of the rodent Oat5s. Thus, the rodent and human forms have been assigned different names within the SLC22A family, with the human form retaining its SLC22A10 systematic name and the rodent Oat5s being designated Slc22a19 [9–12, 34–37]. In rats and mice the Northern blotting and RT-PCR studies showed the presence of Oat5 (Slc22a19) mRNA only in the kidney [9–12], whereas in humans OAT5 (SLC22A10) mRNA was located by Northern blotting exclusively in the liver [34, 35]. Oat5 from mice (mOat5) and rats (rOat5) have been characterized, being at the level of cDNA and peptide sequences identical to 88% and 82%, respectively. rOat5 protein exhibits limited identity to hOAT4 (47%), rOat1 (39%), rOat2 (37%), and rOat3 (37%) proteins [9, 10]. mOat5 was identified as the 85 kDa protein band by Western blotting of renal brush border membrane [12], whereas in the rOat5-transfected HEK293 cells and in renal total cell membranes the protein showed up as the 65 kDa and 72 kDa bands, respectively [10, 38]. rOat5 mRNA was localized to the proximal tubule S2 and S3 segments (S2 < S3) in isolated tubules by RT-PCR [10]. By immunohistochemistry of the rat and mouse kidneys, the transporter was detected in the brush border membrane of proximal tubule straight segment (S3) [10, 12]. Oat5 has been defined as a probenecid-sensitive organic anion/dicarboxylate exchanger, which can transport ochratoxin A, dehydroepiandrosterone sulfate, and estrone-3-sulfate and be inhibited by bumetanide, furosemide, penicillin G, sulfobromophthalein, and by some sulfate but not glucuronide conjugates [10]. Moreover, mOat5, but not rOat5, was found to transport salicylate, but none showed a significant affinity for p-aminohippurate [9–12]. Breljak et al. [38] have recently described that renal expression of Oat5 in rats and mice exhibits the female-dominant sex differences. Studies carried out in our laboratory have shown modifications in the abundance of Oat5 protein in homogenates and in apical membranes from total renal tissue in different models of renal injury [13–15]. Moreover, our group has been pioneer in the detection of Oat5 in urine [13]. This allowed us to evaluate Oat5 renal excretion in the presence of different levels of renal damage, associated with different periods of ischemia and renal reperfusion and with the induction of nephrotoxicity by using different doses of mercuric chloride or cisplatin. In these studies it was observed that Oat5 renal excretion is increased even when there were no changes in the conventionally used biomarkers of renal injury as uraemia, creatininemia, and urinary AP activity [13–15]. Therefore, Oat5 renal excretion has been proposed as a biomarker for early renal damage.

It is important to make a reference about the utility of renal damage traditional biomarkers in this work: the urea is the main nitrogenous waste product of proteins metabolism. It is synthesized in the liver and excreted at renal level through glomerular filtration with variable absorption and secretion. Its increase in blood could be caused by an acute or chronic renal failure or a decrease of renal perfusion. Although plasma urea is a frequent parameter used in the assessment of renal function, it is low sensitive and only rises when more than a half of renal function is lost, and it is not very specific. It can also rise in blood due to other factors such as an important increase of protein contribution or an important increase of protein catabolism. Creatinine is a product of protein metabolism which is derived from creatine. Creatine is stored in the muscle as creatine and phosphocreatine before being metabolized and released to circulation as creatinine. Creatinine is eliminated by glomerular filtration, it is not reabsorbed, and its tubular secretion is minimal. Validity of creatinine as a proof of renal function is based on the premise that, in a normal individual in stable conditions, as creatinine is released from muscular deposits at a constant rate during the day, its accumulation in the body is prevented by a renal excretion mechanism. The most important cause of the increase of this metabolite in plasma is an alteration of the glomerular filtration. Nevertheless, it may also rise through an elevated pathologic muscular change or in individuals with an elevated muscle mass [39]. In a normal kidney only a small quantity of low molecular weight proteins filtrates and most of them are reabsorbed in tubules. The presence of high amounts of proteins in urine could be a sign of an important renal disease, even preceding other symptoms [19, 20]. Nevertheless benign proteinuria could appear in case of fever, cold exposure, intense physical work, and so forth. AP is an enzyme expressed in the apical membrane of tubular cells, with predominance on the brush border of the proximal tubule cells [40]. An increase of urinary excretion of this protein involves damage in brush border membrane with loss of microvilli structure [41]. Histopathological studies are well known for them usefulness in the diagnosis of kidney and other organs diseases, but they have the disadvantage of being invasive methods, which complicates their use especially on humans.

After having described that the increase of Oat5 in urinary excretion, in renal failure models studied [13–15], precedes modifications on the traditional parameters previously mentioned, we wonder if Oat5 renal excretion would be modified in pathologies of nonrenal origin but associated with some type of renal nephropathy. Therefore, the objective of this work was to evaluate the Oat5 urinary excretion and its renal expression in a model of vascular calcification, which according to our laboratory shows a decrease in the glomerular filtration rate, with tubular damages, histological alterations and without modifications on the uraemia [4–8]. Oat5 urinary excretion in this model was compared with other traditional markers of renal damage such as creatinine in plasma, proteins in urine, and alkaline phosphatase activity in urine. None of these parameters were modified in this vascular calcification model. On the contrary, Oat5 urinary excretion, evaluated by Western blotting technique and normalized with the concentration of creatinine in urine, was increased by 111%. The initial centrifugation of urine allowed us to affirm that urinary Oat5 was not due to the presence of tubular cells from cellular shedding. To evaluate the origin of urinary Oat5, its renal expression was evaluated by immunohistochemistry studies and Western blotting in total homogenates and apical membranes. 

An increase of Oat5 abundance both in homogenates and apical membranes from renal tissue of animals with vascular calcification was observed. The increase of Oat5 protein in homogenates could indicate an increase in the synthesis of this protein or a decrease of its degradation.

In this experimental model of vascular calcification we have described modifications in the renal transporters expression. The abundance of the Na^+^-K^+^-2Cl^−^ cotransporter (NKCC2) showed an increase in renal medulla homogenates and did not change in renal cortex homogenates [7]. On the other side, Oat1 protein revealed a decrease in its expression in renal cortex homogenates [8]. Heterogeneous changes observed in NKCC2, Oat1, and Oat5 expression in homogenates of renal tissue in animals with this pathology underscore the selectivity of the response.

The fact that the understudy protein also increases in apical membrane, but the relation between Oat5 abundance in apical membranes and homogenates has a value less than unity, suggests a diminished traffic of the protein to the membrane or an increase of its release towards tubular lumen. The increase of Oat5 excretion in urine would be in favour of the latter hypothesis. 

It is known that calcium has an important role in numerous cellular functions [42]. Changes in the metabolism of renal calcium generated, in this model, by vitamin D_3_ overdose [43] could cause the activation of several pathways of intracellular signaling aimed to modify the synthesis and/or degradation of Oat5 protein, its traffic to the apical membrane, and its urinary excretion. It is important to note that until date the cellular mechanisms of physiological regulation of Oat5 are unknown.

Hence, the preclinical animal results showed in this work in addition to the data presented before by our group [13–15] propose that Oat5 urinary excretion might potentially serve as a noninvasive early biomarker of mercury, cisplatin, ischemic, and vascular calcification which induced renal damage. Efforts to identify biomarkers to assist in early diagnosis of renal damage have yielded many promising candidates. To date, most studies have emphasized discovery, characterization, and validation of individual biomarkers using a single experimental model of kidney injury. This approach is necessary in the initial stages of biomarker development; however, translation to general clinical applicability requires considerable additional work [44].

As it was previously described, Oat5 was discovered by in silico analysis of the Ensembl mouse genome database [9]. Although orthologs have been identified in both mice and rats (Slc22a19), Vanwert et al. [37] have postulated the importance of discovering a human orthologue for rodents Oat5. Rat Oat5 has been proposed to act like human OAT4 (hOAT4, SLC22A11) since both transporters share some common characteristics. For example, hOAT4 is also localized to the proximal tubule brush border membranes and also transports ochratoxin A, dehydroepiandrosterone sulfate, estrone-3-sulfate, and various others organic anions [10, 36, 37]. Nevertheless, rOat5 shows a remote amino acid sequence similarity to hOAT4 (47%); rOat5 is expressed mainly in the late segments (S2 and S3) of proximal tubules whereas hOAT4 is expressed throughout the proximal tubules (from S1 to S3); rOat5 does not transport p-aminohippurate (PAH, a prototypical substrate for renal organic anion transport) while hOAT4 transports PAH [10]. At this point, it is important to mention that preliminary studies carried out in our laboratory detected one band with rOat5 antibodies in urine samples from four adult healthy humans with identical molecular weight to that observed in rat urine (M. S. Trebucobich, M. H. Hazelhoff, and R. P. Bulacio; A. Brandoni, and A. M. Torres, *unpublished data*, 2013). Current experiments are performed in our laboratory in order to characterize the nature of the protein detected in human urine with anti-rOat5 antibodies.

## 5. Conclusion

The results obtained in this work indicate that Oat5 urinary excretion in the studied pathology is increased in the absence of modifications of traditional parameters to evaluate the renal function like urea in plasma, creatinine in plasma, proteins in urine, and AP activity in urine. However, the increase in urinary excretion of Oat5 carrier would occur in conjunction with histological alterations and modifications in hemodynamic as well as tubular renal functions as previously described [7]. These last parameters have to be evaluated through the use of invasive methodologies, unlike Oat5 urinary determination. So, it is possible to postulate the urinary excretion of Oat5 as a potential noninvasive biomarker of renal damage associated with vascular calcification.

## Figures and Tables

**Table 1:** Systolic arterial pressure (SAP), mean arterial pressure (MAP), body weight, kidney weight, renal/body weight, total calcium in plasma, urea levels in plasma, creatinine levels in plasma, and total calcium levels in aorta in control and treated (vitamin D_3_ 300,000 IU/kg, b.w, i.m) rats.

	Control (n = 4)	Treated (n = 4)
SAP (mmHg)	100 ± 5	140 ± 8*
MAP (mmHg)	55 ± 3	76 ± 4*
Body weight (g)	363 ± 10	363 ± 3
Kidney weight (g)	2.61 ± 0.11	2.45 ± 0.05
Renal/body weight	0.0072 ± 0.0002	0.0068 ± 0.0001
Plasma calcium (mg/dL)	9.4 ± 0.4	9.8 ± 0.7
Plasma urea (g/L)	0.48 ± 0.06	0.53 ± 0.06
Plasma creatinine (mg/L)	6.2 ± 0.5	6.8 ± 0.8
Total calcium levels in aorta (*μ*mol/g dry weight)	20 ± 3	50 ± 4*

Results are expressed as mean values ± SEM.

*P < 0.05.
